# Supplementary material for: Multi-omics computational analysis unveils the involvement of AP-1 and CTCF in hysteresis of chromatin states during macrophage polarization
Source: Front Immunol. 2023 Dec 20;14:1304778. doi: 10.3389/fimmu.2023.1304778 (PMC10761412; doi:10.3389/fimmu.2023.1304778)
Supplement: Supplementary file 1 [file DataSheet_1.docx]

Supplementary Material

# Supplementary Figures

**Supplementary Figure 1.** Gene expression in log(FPKM) of key genes of M1 (Il6,MyD88,Nfkb1,Nos2,Tnf) and M2 (Arg1,Jak1,Pparg,Retnla) during M0->M1->M0 polarization in 96 hours.

**Supplementary Figure 2.** Gene expression in log(FPKM) of marker genes of M1 (Cd68,Cd80,Cd86,Il12a/Il12b,Il1b, Tlr2, Tlr4) during M0->M1->M0 polarization in 96 hours.

**Supplementary Figure 3.** PCA plot of RNA-seq expression (averaged across three replicates for each sample) during M0->M1->M0 trajectory.

**Supplementary Figure 4.** Gene expression in log(FPKM) of 10 selected M0->M1->M0 hysteresis genes over time.

**Supplementary Figure 5.** Confusion matrix of logistic regression results across each group (Random, PCA, hysteresis genes, and marker genes) within the Andres and Chuan datasets. The x-axis represents the predicted labels and the y-axis represents the true labels. The numbers in the matrix represent cell counts.

**Supplementary Figure 6.** The four graphs represent the contribution of each gene cluster to the differences in the expression between the starting and endpoints of macrophage polarization, as measured by the Manhattan distance. Each graph corresponds to a specific polarization-de-/repolarization trajectory. The y-axis represents the gene clusters and the x-axis represents the contribution of each cluster to the overall difference.

**Supplementary Figure 7.** Gene expression levels of *S100a8* and *S100a9* were measured at different time points during polarization/depolarization. The x-axis represents time and the y-axis represents gene expression levels (log_10_FPKM).

**Supplementary Figure 8.** Changes in Junb motif-binding sites within all ATAC-seq accessible regions of 0 h M0 macrophages and 96 h deM0 macrophages.

**Supplementary Figure 9.** Changes in Junb motif-binding sites within all ATAC-seq accessible regions of 0 h M0 macrophages and 96 h deM0 macrophages.

**Supplementary Figure 10.** scATAC-seq UMAP of different macrophage cell states.

**Supplementary Figure 11.** Density plot of Arg1 (M2 polarization marker) of different macrophage cell states based on scATAC-seq.

**Supplementary Figure 12.** Density plot of Nos2 (M1 polarization marker) of different macrophage cell states based on scATAC-seq.
